# Microtensile Bond Strength Between Zirconia Core and Veneering Porcelain After Different Surface Treatments

**Published:** 2017-11

**Authors:** Sakineh Nikzadjamnani, Simindokht Zarrati, Masomeh Rostamzadeh

**Affiliations:** 1 Associate Professor, Dental Research Center, Dentistry Research Institute, Tehran University of Medical Sciences, Tehran, Iran; Department of Prosthodontics, School of Dentistry, Tehran University of Medical Sciences, Tehran, Iran; 2 Assistant Professor, Dental Research Center, Dentistry Research Institute, Tehran University of Medical Sciences, Tehran, Iran; Department of Prosthodontics, School of Dentistry, Tehran University of Medical Sciences, Tehran, Iran; 3 Assistant Professor, Dental Research Center, Dentistry Research Institute, Tehran University of Medical Sciences, Tehran, Iran; Department of Prosthodontics, School of Dentistry, Kurdistan University of Medical Sciences, Sannadaj, Iran

**Keywords:** Zirconium Oxide, Surface Properties, Dental Stress Analysis, Dental Veneers

## Abstract

**Objectives:**

The long-term clinical success of all-ceramic restorations requires sufficient bond strength between the veneering ceramic and substructure. The present study compared the effects of three methods of surface treatment on the microtensile bond strength of the veneering porcelain to zirconia.

**Materials and Methods:**

Twelve zirconia blocks were randomly divided into four groups of aluminum oxide (Al_2_O_3_) air abrasion, carbon dioxide (CO_2_) laser irradiation, erbium-doped yttrium aluminum garnet (Er:YAG) laser irradiation, and control samples (no surface treatment). After surface treatment, the zirconia blocks were veneered with porcelain. To assess the surface topographies, four surface-treated specimens were left uncoated. Microtensile bond strength was tested in each group and was statistically analyzed by one-way ANOVA and post-hoc Tukey’s test. Surface topographies were examined by using scanning electron microscopy (SEM).

**Results:**

The highest and lowest microtensile bond strength values were recorded in the Al_2_O_3_ (43.6±10.0 MPa) and control groups (34.7±8.2 MPa, P<0.05). The bond strengths in the CO_2_- and Er:YAG-irradiated groups were equal to 40.4±6.5 MPa and 38.2±7.5 MPa, respectively. The majority of the failures (mean=92.44%) were of cohesive nature located in the veneer, followed by mixed fractures (mean=7.6%). The milling marks of the computer-aided design/computer-aided manufacturing (CAD/CAM) machine were apparent in the control samples, while desert-like micro-cracks were observed on the surfaces treated with CO_2_ and Er:YAG lasers. Al_2_O_3_ air abrasion produced the roughest topography.

**Conclusions:**

Al_2_O_3_ air abrasion resulted in a higher microtensile bond strength compared to CO_2_ or Er:YAG laser irradiation. Cohesive failure mode was predominant. No pure adhesive failures were observed.

## INTRODUCTION

The increasing demand for esthetics has increased the popularity of all-ceramic restorations. Many all-ceramic systems have been introduced for fabrication of fixed partial prostheses. The inherent properties of ceramics, such as brittleness, have limited their application, especially in posterior teeth [1]. However, zirconia is a material of choice for the substructure due to its superior mechanical properties, without the limitations related to the size or position of the restoration [1].

Yttrium-stabilized tetragonal zirconia polycrystals (Y-TZP) offer superior biomechanical properties such as stress-induced transformation toughening in addition to optical advantages. These properties make zirconia a suitable alternative to metals in prosthodontics [2–5]. Moreover, the precision and versatility of computer-aided design/computer-aided manufacturing (CAD/CAM) have made zirconia-based materials one of the most reliable options for a wide variety of clinical applications [1, 6–9].

Several studies have investigated the bond strength of zirconia-based veneer ceramics after the use of various surface treatment methods [10–15]. Particle abrasion and silica coating are the most common techniques for surface treatment [10–15]. Although airborne-particle abrasion with aluminum oxide (Al_2_O_3_) may enhance the surface energy, surface area and wettability of the zirconia prior to adhesive cementation, it may adversely affect the flexural strength and reliability of the restoration [16]. Chipping of the veneering ceramic or delamination of the veneer from the core are the most frequently occurring technical complications in core-veneered zirconia restorations. The incidence of chipping has been reported to be 13% to 25% in different investigations [1]. However, the long-term evaluation of zirconia restorations has shown good success rates [4]. The clinical success and reliability of these restorations are highly dependent on the bond strength at the interface between the veneering ceramic and core [1]. The core-veneer bond strength is influenced by several variables such as the coefficient of thermal expansion (CTE) of the ceramics (core and veneer) and wetting properties [1, 5]. Various surface treatment methods have been used for increasing the surface area to achieve stronger bonding and to decrease the rate of chipping of the veneering porcelain [1, 9]. Although many researchers have recommended airborne-particle abrasion of zirconia surface prior to veneering, others have stated that this treatment is destructive and not beneficial [1, 9]. Controversies also exist with regard to the effect of lasers on zirconia [1]. The purpose of the present study was to compare the microtensile bond strength at the core-veneer interface of zirconia restorations after using carbon dioxide (CO_2_) laser irradiation, erbium-doped yttrium aluminum garnet (Er:YAG) laser irradiation and Al_2_O_3_ airborne-particle abrasion for surface treatment of zirconia.

## MATERIALS AND METHODS

An aluminum mold with the dimensions of 12×12× 4 mm^3^ was prepared and mounted in a holder. After being covered with Cercon scanning powder (DeguDent GmbH, Frankfurt, Germany), the mold was scanned with Cercon Brain scanner (DeguDent GmbH, Frankfurt, Germany). The scanned model was used to fabricate 12 cuboids of the same size from 6×2.5×2.5 cm^3^ non-sintered zirconia cylindrical blocks (Cercon, DeguDent GmbH, Frankfurt, Germany).

### Surface treatment methods:

**Fig. 1:**
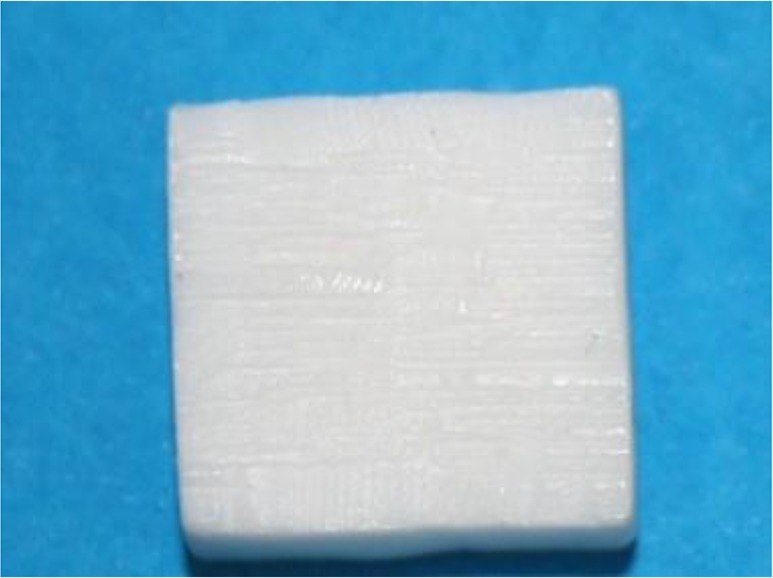
Zirconia blocks prepared for surface treatment

Twelve zirconia cylinders were randomly divided into four groups according to the surface treatment method (Fig. 1): Group 1 (control): CAD/CAM-milled surfaces without surface treatment. Group 2: The CO_2_ laser (Smart US20D, DEKA M.E.L.A. Srl, Florence, Italy) was used at the 10600-nm wavelength and output power of 5W in the continuous-wave mode for 10 seconds. Group 3: Al_2_O_3_ airborne-particle abrasion was performed using a sandblast machine (Beco Industries, China) with 110μm particle size for 15 seconds at a 10mm distance from the surface and with a pressure of 3.5 bar.

Group 4: The Er:YAG pulsed laser (Laser Smart 2940, DEKA M.E.L.A. Srl, Florence, Italy) was irradiated at the 2.94-μm wavelength and 300mJ/pulse energy for 2 minutes, with a focal distance of 5mm and frequency of 10Hz. Intermittent cooling with air and water spray was used throughout the irradiation. The prepared cuboids were cleaned in an ultrasonic cleaner (BioSonic UC50D, Coltene/Whaledent AG, USA) for 5 minutes. The specimens were also steam-cleaned (Steam Cleaner, VAP 6, Zhermack, Italy).

### Application of porcelain:

**Fig. 2:**
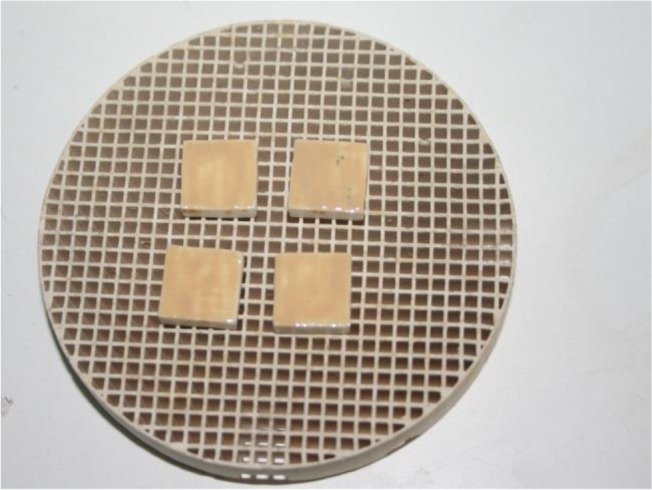
Application of liner porcelain on the samples

One layer of liner porcelain (Cercon Ceram Kiss Liner, D4 shade; DeguDent GmbH, Frankfurt, Germany) was applied to the cuboids (eight surface-treated cuboids were covered with porcelain, while four surface-treated specimens were left uncoated to study the surface topography). The specimens were then fired in a ceramic furnace (VITA Vacumat 6000 M premium, Vident, CA, USA) according to the manufacturer’s instructions (Austromat 3001, Dekema Dental-Keramiköfen GmbH, Freilassing, Germany). The D4 liner shade was selected since it creates a strong contrast between the liner and overlying porcelain, which facilitates the visual differentiation of the probable fracture line (Fig. 2). The margin porcelain powder (Cercon Ceram Kiss, DeguDent GmbH, Hanau, Germany) was then mixed with an appropriate amount of liquid and was applied as a single layer to the prepared surface of each cuboid. The specimens were fired again in the VITA furnace. The firing of the veneering porcelain was done in two steps according to the manufacturer’s instructions. The proper height of 4mm was obtained by using a 12×12×8 mm^3^ aluminum cylinder as a condensing mold. The height of the fired porcelain was slightly more than 4mm to compensate for the firing shrinkage (Table 1). A special metal mold was used to prepare the specimens for sectioning. The mold was sealed at both ends. A three-component resin (AMPSET, Cold Mounting Systems, Turkey) was then mixed and poured into the mold until the mold’s base was covered. The primary setting time was 15 to 20 minutes with a final setting time of 24 hours. Cyanoacrylate adhesive (Mitreapel, Beta Chemical Industry, Istanbul, Turkey) was then used to secure the specimens inside the resin base to prevent mobility while the rest of the mold was being filled with the three-component resin. The specimens were removed from the molds after the setting of the resin. The samples were then fixed inside the sectioning machine (Mecatome P100, Presi, Grenoble, France), and were sectioned by a rotating diamond-coated disc under cold water irrigation. The sections were made in two steps: 1mm cross-sections were first prepared and the specimens were remounted to obtain microbars with the dimensions of 1×1×6 mm^3^. The microbars of the first row of sections were excluded due to the of possibility defects. The resultant microbars were bathed in acetone for 5 minutes inside the ultrasonic cleaner, were washed under running hot water and were dried.

**Table 1.** Porcelain firing cycles according to the manufacturer’s instructions

	**Standby temp. (°C)**	**Final temp. (°C)**	**Drying time (min)**	**Heating-up time (min)**	**Hold-time (min)**	**Vacuum-hold time (min)**
Paste-Liner	575	970	9	6	1.00	6
Margin 1	450	850	9	6	1.00	6
Dentine 1	450	830	9	6	1.30–2.30	6

**Fig. 3:**
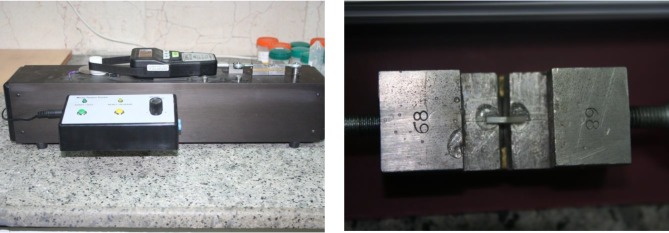
(A) Microtensile bond strength tester machine. (B) The sample in the microtensile bond strength tester machine

The dimensions of the microbars were examined using a digital caliper. The veneer-core interface was inspected under a stereomicroscope (SZX9, Olympus Optical Co., Tokyo, Japan) at ×40 magnification. Seventeen sound microbars from each group were chosen for microtensile bond strength testing. The microbars were fixed to the opposing arms of the microtensile bond strength tester (Bisco Inc., Schaumburg, IL, USA) using Mitreapel cyanoacrylate glue, parallel to the long axis of the arms with the core-veneer interface facing forward. The adhesive was applied to secure the microbars (Fig. 3. A,B). A tensile load was applied to the microbars at a speed of 1mm/minute and the maximum load (N) upon fracture was recorded.

**Fig. 4:**
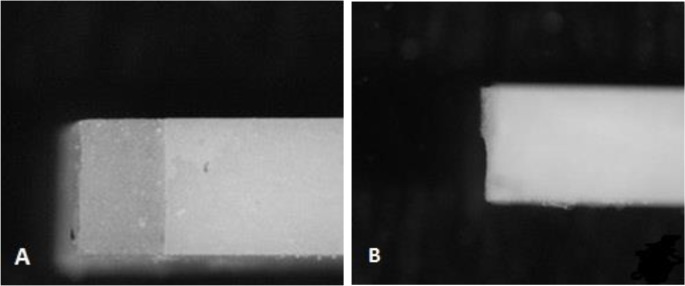
(A) Stereomicroscopic photograph of the cohesive failure in the veneering porcelain, (B) Mixed failure mode

This value was divided by the surface area of the microbars, 1×10^−6^ m^2^, to calculate the bond strength in MPa (N/mm^2^). The fractured microbars were removed from the testing machine, and the fracture sites were observed under the stereomicroscope (Fig. 4). To assess the surface topographies, the four uncoated cuboids (without porcelain veneering) were examined under a scanning electron microscope (SEM, CamScan, MV2300, Oxford, England) at ×100, 250 and 500 magnifications. Also, SEM analysis of the elements was performed on the surfaces with mixed fracture patterns in all groups.

### Statistical analysis:

One-way ANOVA was used for the detection of the possible statistically significant differences in the mean±standard deviation (SD) of the microtensile bond strength values of the four study groups. Post-hoc Tukey’s test was employed for pairwise comparisons. Statistical analysis was performed using PASW statistics 18 software (SPSS Inc. Chicago, IL, USA). A significance level of α=0.05 was set.

## RESULTS

### Microtensile bond strength:

The highest mean microtensile bond strength value was measured in the AL_2_O_3_ air-abrasion group (43.5±10.02 MPa), while the lowest value was measured in the control group (34.7±8.15 MPa). The microtensile bond strengths in the CO_2_ and Er:YAG laser-irradiated groups were equal to 40.3±6.84 MPa and 38.2±7.52 MPa, respectively. The results of one-way ANOVA proved that the difference between the groups was significant (P=0.022). Tukey’s test showed a statistically significant difference between the mean microtensile bond strengths of the Al_2_O_3_ air-abrasion group and the control samples (P=0.014).

Multiple comparisons of the other groups showed no significant differences (P≥0.203, Tables 2 and 3). The mean and SD of the microtensile bond strength values of the groups are shown in Table 3.

**Table 2.** Microtensile bond strength (MPa) of the microbars

**Sample**	**Control**	**Er:YAG**	**CO_2_**	**AL_2_O_3_**
1	41.20	30.10	34.80	34.00
2	50.20	31.20	43.90	35.10
3	42.10	31.60	46.30	40.00
4	42.50	40.70	38.30	46.70
5	39.10	50.60	44.70	44.30
6	32.80	39.60	40.00	56.20
7	40.00	36.80	36.00	49.40
8	23.80	46.30	31.70	42.70
9	29.70	31.70	46.30	28.10
10	44.00	31.80	27.70	30.10
11	33.60	34.60	51.80	29.00
12	30.40	31.70	32.80	50.20
13	21.10	32.80	37.40	45.10
14	22.50	54.60	36.80	51.80
15	30.00	40.00	47.10	41.10
16	32.00	38.70	50.50	55.00
17	36.10	47.10	40.30	62.10

Er:YAG=Erbium-doped Yttrium Aluminum Garnet, CO_2_=Carbon dioxide, Al_2_O_3_=Aluminum oxide

**Table 3.** Descriptive values of the microtensile bond strength in the groups according to the surface treatments (Tukey's test)

**Groups**	**Minimum (MPa)**	**Maximum (MPa)**	**Mean±SD (MPa)**
Control	10.39	50.2	34.7±8.15^a^
Er:YAG	30.1	54.6	38.22±7.52^ab^
CO_2_	27.7	50.5	40.37±6.84^ab^
AL_2_O_3_	10.41	56.2	43.58±10.02^b^

Er:YAG=Erbium-doped Yttrium Aluminum Garnet, CO_2_=Carbon dioxide, Al_2_O_3_=Aluminum oxide, SD=Standard Deviation. The means with different letters

(a–b) indicate significant differences (P<0.05)

### Modes of failure:

No pure adhesive failure was observed in any of the study groups.

**Fig. 5:**
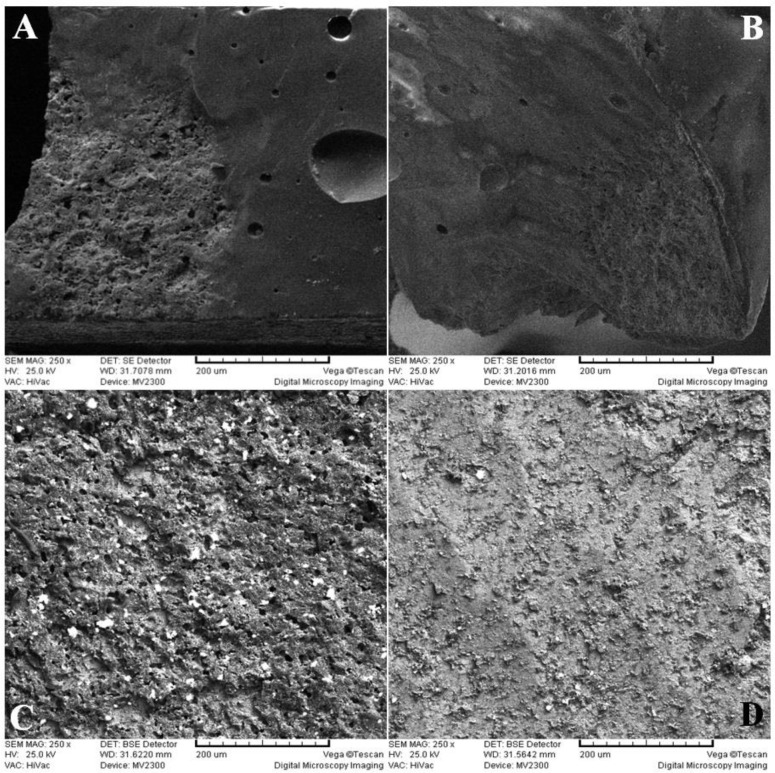
SEM microphotographs of the fractured surfaces: (A) Al_2_O_3_ air abrasion, (B) CO_2_ laser irradiation, (C) Er:YAG laser irradiation, (D) Control group. Note the mixed fracture patterns in all the study groups

The majority of the failures (mean=92.44%) were cohesive and were detected within the veneer adjacent to the core-veneer interface. The mean rate of mixed failure pattern was 7.6% (Table 4, Fig. 4 and 5). Chemical analysis by SEM was performed on the surfaces exhibiting the mixed fracture pattern, which indicated the presence of zirconia and the elements of the veneering porcelain.

**Table 4.** Frequency of the failure modes

	**Cohesive (%)**	**Adhesive (%)**	**Mixed (%)**	**Total (%)**
**Control**	15 (88.2)	0	2 (11.8)	17 (100)
**Er:YAG**	16 (94)	0	1 (6)	17 (100)
**CO_2_**	16 (94)	0	1 (6)	17 (100)
**Al_2_O_3_**	16 (94)	0	1 (6)	17 (100)
**Total**	63 (92)	0	5 (8)	68 (100)

Er:YAG=Erbium-doped Yttrium Aluminum Garnet, CO_2_=Carbon dioxide, Al_2_O_3_=Aluminum oxide

### Topography:

**Fig. 6:**
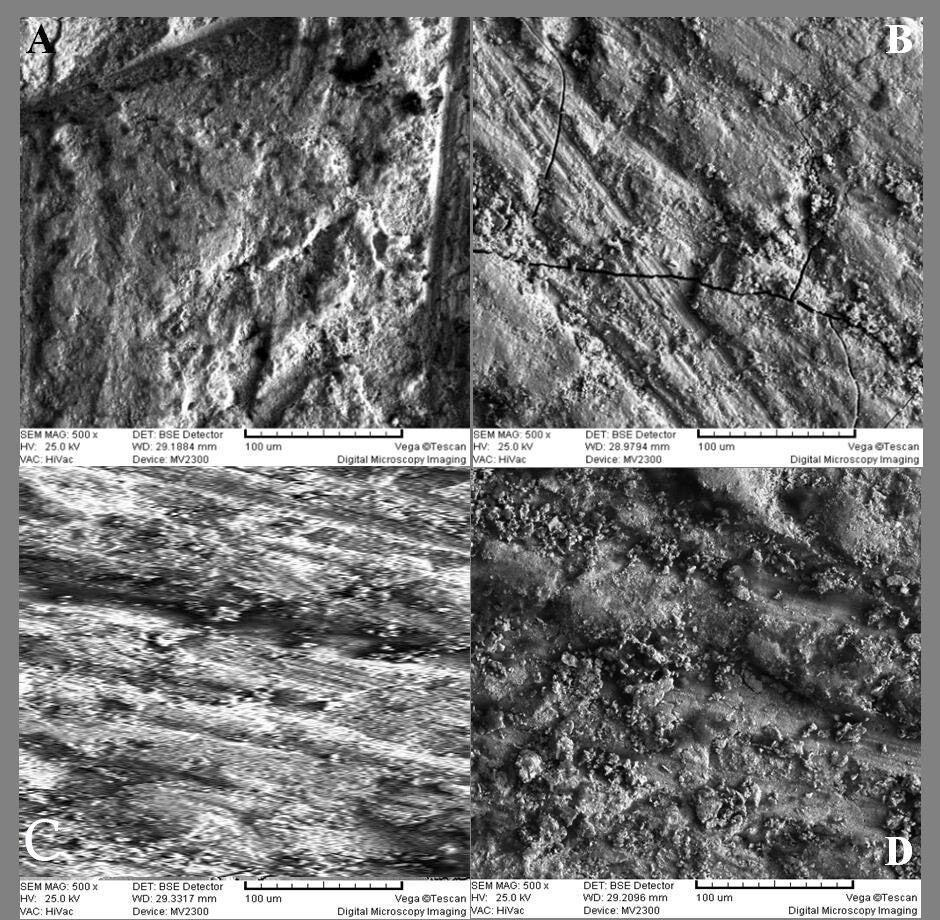
SEM topography microphotographs (×500 magnification), (A) Al_2_O_3_ air abrasion, (B) CO_2_ laser irradiation, (C) Er:YAG laser irradiation, (D) Control group. Note the cracks in the melted surface (decreased micro-roughness) of the laser-irradiated groups and the abrasion lines in the air-abraded group

The control group only showed the milling lines of the CAD/CAM machine over the surfaces. In the Er:YAG-irradiated group, the surface of zirconia was melted and showed desert-like micro-cracks. The same pattern was observed on the surfaces treated with the CO_2_ laser but with more noticeable cracks. The surfaces treated with Al_2_O_3_ particles showed the roughest topography (Fig. 6).

## DISCUSSION

The purpose of the present study was to evaluate the efficacy of three different surface treatments of zirconia core, and to compare the bond strength at the zirconia core-veneering ceramic interface among the treated surfaces. The studies on the bond strength of core-porcelain veneer have mostly used shear strength tests, 3-point/4-point flexural tests, or biaxial flexural tests, which are associated with the structural failure of the specimens [11, 17–19]. The samples used in the shear test settings receive uneven stress during loading. The vertical direction of the load relative to the core-veneer bonding interface and the small size of the tested microbars reduce the chance of structural flaws; however, this method of bond strength testing requires great attention, as the technique is very sensitive and time-consuming [12, 13, 17]. Therefore, application of microtensile bond strength test on dental ceramics needs careful handling of the specimens to avoid creation of structural defects [11]. Al_2_O_3_ airborne-particle abrasion significantly improved the bond strength compared to the control and Er:YAG-irradiated groups. It should be mentioned that the microtensile bond strength in the CO_2_ laser-irradiated group was 40.3±6.84 MPa, almost as high as that of the Al_2_O_3_ airborne-particle abraded group; however, it showed no statistically significant difference from the other groups. Tukey’s test showed a significant difference between the airborne-particle abraded and control groups. Additional studies with different laser energies are suggested. The SEM photomicrographs confirmed the superior quality of the surface topography of the Y-TZP ceramic treated with sandblasting. Other authors have also confirmed an increase in the bond strength with this surface treatment method [1, 9, 20]. It is believed that airborne-particle abrasion increases the micro-roughness and available surface area and improves the surface wettability of the specimens.

However, some authors have described that the micro-porosities formed as a result of surface treatments may act as the initiation point of crack and may increase the fracture probability of the material [1, 21]. Matani et al [1] used airborne-particle abrasion with 80μm particles to decrease the damage to the zirconia surface that they believe would occur with larger particles. Silica coating has also been shown to cause damage to the zirconia surface [1]. Nonetheless, it was precluded in our study because of the limitations in its application and controversies about the effect of this treatment. The effect of these surface treatments on the failure rate of the core-veneer interface needs further assessment by studies of a longer duration [1]. Er:YAG laser evaporates the water content of the lased surface; also, micro-explosions result in the formation of micro-porosities and increased micromechanical retention. On the other hand, the alternate warming and cooling phases cause internal tension in the material, which is likely to negatively affect its mechanical properties.

The high laser energy may also deteriorate the crystal or matrix phase. It is therefore suggested to use low-level lasers and water spray [1, 15, 22].

Gökçe et al [23] found that surface treatment of IPS Empress II ceramic with 5.99% fluoric acid for 5 minutes and with Er:YAG laser (300mJ) resulted in the highest levels of bond strength compared to 600 and 900mJ laser energies and the control group. Since zirconia is water-free and has a white opaque color, the specimens have been coated with a graphite layer in some studies to enhance the absorption of Er:YAG laser energy [1, 24]. In the present study, 300mJ laser energy was used with water spray to eliminate the heating effect of the laser. The obtained bond strength in the Er:YAG laser-irradiated group was lower than those of the CO_2_ laser-irradiated and AL_2_O_3_ air-abraded groups, which is thought to be related to the inherent reflective property of the ceramic and the low level of laser energy absorption within its surface.

No significant difference was found between the laser-irradiated groups and the control group, which may be attributed to the low laser energies used in this study. However, the average amounts of microtensile bond strength were higher than those in the control group. CO_2_ laser application enhanced the bond strength between the zirconia ceramic and resin cement. The veneering porcelain remained on the zirconia surface in all the microbars and delamination did not occur. It can be concluded that the bond strength between the zirconia and veneering porcelain is higher than the cohesive strength of the veneering porcelain.

Therefore, the veneering porcelain is the weakest point of the restoration rather than the core-veneer interface. Aboushelib et al [10] stated that more than 90% of the specimens showed the adhesive failure pattern. Their materials and methods, however, were different from ours. A significant difference was found between the mean microtensile bond strength values of the AL_2_O_3_ air-abraded group and the control group. No significant difference was found between the bond strengths of the Er:YAG and CO_2_ laser-irradiated groups and the control group; however, all the experimental groups demonstrated a higher roughness and microtensile bond strength than the control group. These findings were in accordance with previous studies [1, 9, 23, 24]. SEM examinations showed that in the control group, the only surface alteration was the milling lines of the CAD/CAM device. Er:YAG and CO_2_ lasers, however, caused significant alterations in the form of melted areas with micro-cracks. The maximum surface micro-roughness was attained after Al_2_O_3_ airborne-particle abrasion. Cavalcanti et al [24] also detected a higher micro-roughness after airborne-particle abrasion using 50μm alumina compared to the control group. They also noticed that the laser-irradiated surfaces show melted areas with micro-cracks [24]. With increased laser energy at the surface, the size of the cracks also increases. The SEM findings of the present study are similar to that of the studies which found CAD/CAM preparation lines on the zirconia [10, 11]. It has been found that surface treatment with Al_2_O_3_ sand blasting replaces these lines with a microscopically rough surface. Al_2_O_3_ sand blasting improved the bond strength in the present study, probably due to the improved surface micro-roughness, which is thought to enhance the micromechanical bonds and increase the available surface area for the chemical bonds, associated with a much higher bond strength and a significantly lower failure rate [24, 25]. Arami et al [9] found a similar surface roughness after the use of Er:YAG laser and air abrasion, and also reported that neodymium-doped yttrium aluminum garnet (Nd:YAG) and CO_2_ lasers could destruct zirconia. The difference between their findings and our results might be due to the different laser energies and experimental settings.

Matani et al [1] reported a higher monoclinic content after airborne-particle abrasion and laser irradiation than that in the experimental group (experimental glass slurry); however, the difference was not statistically significant. It is believed that the monoclinic layer is not beneficial for veneering [1].

## CONCLUSION

Within the limitations of the present study, the following conclusions can be drawn:

1- All the zirconia surface treatment methods resulted in an increase in the bond strength to the overlying ceramic compared to the control group; however, the microtensile bond strength of the air-abraded group was significantly higher than that of the laser-treated groups and control group (P=0.014).
2- The laser-irradiated groups were not significantly different from the control group in terms of the microtensile bond strength (P≥0.203).
3- The air-abraded group showed a higher roughness than the other groups.
4- No pure adhesive failure mode was seen in the groups (92.44% cohesive failure in the veneering porcelain and 7.6% mixed failure).
5- Most fractures were cohesive and were located in the veneering porcelain adjoining the interface, indicating that the most fragile part of the restoration is the veneering porcelain.
